# Mind the Gap: The Effects of Temporal and Spatial Separation in Localization of Dual Touches on the Hand

**DOI:** 10.3389/fnhum.2018.00055

**Published:** 2018-02-13

**Authors:** Renata Sadibolova, Luigi Tamè, Eamonn Walsh, Matthew R. Longo

**Affiliations:** ^1^Department of Psychological Sciences, Birkbeck, University of London, London, United Kingdom; ^2^Institute of Psychiatry, Psychology, and Neuroscience, King’s College London, London, United Kingdom

**Keywords:** tactile localization, funneling, lateral inhibition, body representation, somatosensory cortex

## Abstract

In this study, we aimed to relate the findings from two predominantly separate streams of literature, one reporting on the localization of single touches on the skin, and the other on the distance perception of dual touches. Participants were touched with two points, delivered either simultaneously or separated by a short delay to various locations on their left hand dorsum. They then indicated on a size-matched hand silhouette the perceived locations of tactile stimuli. We quantified the deviations between the actual stimulus grid and the corresponding perceptual map which was constructed from the perceived tactile locations, and we calculated the precision of tactile localization (i.e., the variability across localization attempts). The evidence showed that the dual touches, akin to single touch stimulations, were mislocalized distally and that their variable localization error was reduced near joints, particularly near knuckles. However, contrary to single-touch localization literature, we observed for the dual touches to be mislocalized towards the ulnar side of the hand, particularly when they were presented sequentially. Further, the touches presented in a sequential order were slightly “repelled” from each other and their perceived distance increased, while the simultaneous tactile pairs were localized closer to each other and their distance was compressed. Whereas the sequential touches may have been localized with reference to the body, the compression of tactile perceptual space for simultaneous touches was related in the previous literature to signal summation and inhibition and the low-level factors, including the innervation density and properties of receptive fields (RFs) of somatosensory neurons.

## Introduction

The brain maintains maps of body parts which reflect an orderly organization of the somatosensory system with adjacent areas on the skin represented by adjacent neurons in the cortex (Penfield and Boldrey, 1937; Kaas et al., 1979; Sereno and Huang, 2006). Given this somatotopic arrangement, a touch on the skin results in activation at a corresponding location of the somatotopic map, which should in theory give rise to an accurate percept of the touch at a specific location of the body, which need only be “read off” from the location of the activation. Nevertheless, the literature reports that the touches may be mislocalized by patients (Förderreuther et al., 2004; Anema et al., 2009), and healthy adults (Mancini et al., 2011; Margolis and Longo, 2015). Further, the early somatotopic maps are considerably distorted due to cortical magnification factors (Penfield and Boldrey, 1937), which impacts on a perception of the distance between two touches. The secondary processes are therefore required to alleviate these distorting influences in tactile distance perception, and in mapping of the perceived touch on the skin.

Several literature sources posit an existence of a secondary body representation in the brain which exerts a “top-down” influence by ameliorating the impact of the distorted somatotopic maps (Longo et al., 2010; Medina and Coslett, 2010; Longo, 2015). Head and Holmes (1911) first introduced a concept of a “superficial schema” to explain their findings with patients with *atopognosia*. These patients could detect that they had been touched suggesting that the primary representations of touch was preserved. However, their inability to identify where this touch occurred was thought to indicate deficits in some additional processing stage underlying the localization of perceived touch on the skin (Head and Holmes, 1911). Although the serial model of the tactile detection and localization which this logic implies was later disputed (Harris et al., 2004, 2006; Medina and Coslett, 2016), the existence of the secondary body representation is widely accepted and has been corroborated by abundant evidence (see reviews by Longo et al., 2010; Medina and Coslett, 2010).

Longo et al. (2010) recently suggested a model whereby the localization of touch initially takes place within primary somatotopic maps followed by a subsequent mapping onto a body representation in secondary somatosensory areas. Tactile localization across these processing stages may be studied by examining errors in tactile performance. For instance, Rapp et al. (2002) described two patients with left hemispheric damage who showed consistency in perceived tactile locations with respect to one another while exhibiting deficits in their overall mapping on the body surface (Rapp et al., 2002). These distinct classes of localization error imply a preserved somatotopy but an impaired mapping of the perceived touches onto a secondary representation of tactile perceptual space, respectively. To study the systematic displacement of touch on skin by healthy individuals, Mancini et al. (2011) developed a paradigm wherein participants mark the perceived tactile locations on a featureless size-matched silhouette of the limb (e.g., hand) on a computer screen. In the analysis stage, the responses are aligned in a common coordinate space with the actual locations on the skin in order to compute the systematic displacement of perceived touches relative to their actual locations, i.e., the *constant* localization error. The *variable* localization error quantified as a response variability, i.e., the spread of localization attempts, may also be determined in this paradigm. The evidence showed for the touch on the hand dorsum to be mislocalized in distal and radial directions, i.e., towards fingers and the thumb, respectively (Mancini et al., 2011). These mislocalizations were similar across somatosensory modalities, suggesting a supra-modal representation, and they were observed irrespective of the response modality (Mancini et al., 2011).

Yet another instance in which the tactile location is systematically misjudged is a mere presence of another touch (Green, 1982). Intriguingly, Green (1982) reports a difference in localization errors on the arm for single touches which tend to be biased towards the joints with an increasing joint proximity of the touch (see also Cholewiak and Collins, 2003), and for dual touches which are mislocalized in relation to each other (Boring, 1942; Von Békésy, 1975; Green, 1982). Specifically, Green reports a compression of perceptual space between dual touches, both in the localization and tactile distance perception tasks. While the literature on actual localization of dual touches is scarce, there is an abundance of evidence on perception of their spatial separation, i.e., of tactile distance. The “low level” factors in the periphery and primary somatosensory cortex (S1) are widely discussed to account for the distortions in tactile distance perception given its link to tactile spatial acuity (Weber, 1996; Taylor-Clarke et al., 2004; Anema et al., 2008; Longo and Haggard, 2011; Longo and Sadibolova, 2013; Miller et al., 2016).

In brief, touch is mediated by four classes of mechanoreceptors in the skin. Two receptor types which respond to a steady pressure are classified as slowly-adapting (SA), while the other two receptor types sensitive to tactile motion are fast-adapting (FA; Johansson and Vallbo, 1979; Johansson et al., 1980). In the studies discussed here, participants make judgments about points of touches on their skin, which together with the edges and corners stimulate predominantly the Merkel cell receptors (SA1 fibers; Johnson, 2001). When a stimulus pair is placed on the skin, the touches will feel as distinct points if they stimulate non-overlapping SA1 and RA1 receptors. However, they will not be discernible if they are contained within the same receptive field (RF; Johansson and Vallbo, 1979). Thus, the innervation density, RF properties of tactile neurons and a degree of cortical magnification affect tactile spatial sensitivity (Johansson and Vallbo, 1979; Brown et al., 2004). Sensitivity is highest at fingertips which are densely populated by neurons with small RFs (Johansson and Vallbo, 1979) and have a large cortical representation (Penfield and Boldrey, 1937). Conversely, it deteriorates at more proximal body parts (Weinstein, 1968; Mancini et al., 2014) with smaller representations, decreased innervation density, and larger RF size and overlap (Penfield and Boldrey, 1937; Sur et al., 1980).

Correspondingly, Von Békésy (1975) reported that the perceived tactile distance increases monotonously from zero (tactile spatial acuity threshold) with the increasing stimulus distance. This is corroborated in Weber’s illusion where the same tactile distance feels smaller on less sensitive skin surfaces with higher acuity threshold (Weber, 1996; Anema et al., 2008; Longo and Haggard, 2011). Further, this literature overlaps with the “funneling” effect (Von Békésy, 1958, 1975; Chen et al., 2003; Barghout et al., 2009), reporting a mislocalization of simultaneous touches towards each other, be it on a continuous skin surface or on fingers. Moreover, there are reports of a mislocalization bias towards a touch of the stimulus pair which was applied with a greater pressure (Von Békésy, 1975; Barghout et al., 2009). The processes of summation and inhibition of tactile signals are implicated in these studies. For instance, the response patterns for two near-threshold simultaneous touches are “summated”, which results in an exaggeration of the center of the stimulus pattern and a reduction at the sides (actual locations) due to an inhibitory RF surround of each other cell (Von Békésy, 1975; Chen et al., 2003). The literature suggests that, rather than being a peripheral phenomenon, the funneling effect has a central (S1) origin (Gardner and Spencer, 1972; Chen et al., 2003).

Stimulations outside the excitatory center of the RF reduce neuronal responses in the RF due to its inhibitory surround. The neuronal refractory period, and additional feedforward and feedback interneuron connections also account for a response suppression of successive touches (Gardner and Johnson, 2000). Behavioral studies report increasingly impaired tactile performance, e.g., the detection (Gardner and Costanzo, 1980; Tamè et al., 2015) of a target stimulus when a masking stimulus is presented in a window of ~500 to −500 ms from the onset of the target stimulus. Accordingly, the neurophysiological evidence (Angel, 1969) shows that when the interval between two successive tactile stimuli is less than ~500 ms, the probability of discharge of single thalamic cells is a monotonic function of the interval between stimuli down to 40 ms, at which point the second stimulus is “masked”, i.e., elicits no response. The temporal separation of dual stimuli indeed plays a great role in a perception of their spatial separation. Under the right temporal conditions (usually less than ~300 ms), the spatial mislocalization of stimuli may even induce illusory tactile movement on the skin (Geldard and Sherrick, 1972; Barghout et al., 2009). In the Tau effect, a person given two successive spatial intervals, automatically equalizes the ratios of their temporal presentation with the ratios of their perceived distance. Thus, the perceived spatial separation may increase with increased temporal separation (Helson, 1930; Jones and Huang, 1982; Goldreich, 2007).

The aim of this study was to complement and relate the two independent streams of literature, one investigating the systematic errors and precision in localization of single touches, and the other investigating tactile distance perception between dual touches. To do so, we adapted the procedures from single touch localization studies by Mancini et al. (2011) and Margolis and Longo (2015). Participants were instructed to localize pairs of tactile stimuli on the dorsum of their hands by indicating the corresponding locations on a size-matched hand silhouette. We chose a sufficient stimulus intensity (60 g) for a clear tactile detection. Two simultaneous touches at the distance lower than that of a spatial acuity threshold may feel as a single touch (Von Békésy, 1975), which is a percept reported for touches less than ~2 cm apart on the hand dorsum (Weinstein, 1968; Mancini et al., 2014). The touches in this study were separated by 2 cm, 3 cm, or 4 cm. To prevent the Tau effect, they were presented in separate blocks of trials either simultaneously or sequentially (1-s delay). The temporal delay was sufficient to reduce the influence of low-level factors associated with the physiology and organization of the somatosensory system while at the same time the memory trace of the first stimulus of the pair was preserved. This study design thus allowed us to investigate the role of spatial and temporal separation in localization of dual touches on the hand dorsum. Additionally, we explored the role of landmarks such as knuckles and a wrist by sub-dividing the dorsum into four regions on the proximo-distal axis. The regions close to joints allowed us to explore the influence of these landmarks in dual-touch localization (Green, 1982).

## Materials and Methods

### Participants

Thirty individuals naïve to the purpose of the study participated (19 females, 29.8 ± 13.6 years). All participants were predominantly right handed, as assessed by the Edinburgh handedness inventory (Oldfield, 1971; Mean ± SD: 77.6 ± 47.5). This study was carried out in accordance with the recommendations of “Guidelines on research ethics”, Department of Psychological Sciences Research Ethics Committee at Birkbeck, University of London. All subjects gave written informed consent in accordance with the Declaration of Helsinki. The protocol was approved by the Department of Psychological Sciences Research Ethics Committee at Birkbeck, University of London. Five photographs were not saved due to a technical issue (camera malfunction) and thus only the data of 25 participants are included in tactile localization analyses.

### Apparatus and Procedure

Participants sat in front of a computer monitor (15 × 12 inches). Their hands and forearms rested on the table with the palms facing down. Both arms were occluded by two sheets of black foamboard running along the sagittal plane of body at the left shoulder and along the transverse plane of the body ~15 cm above the table. The experimenter, seated to participant’s left behind the occluder wall, was facing another computer monitor (22 × 14 inches).

**Figure 1:**
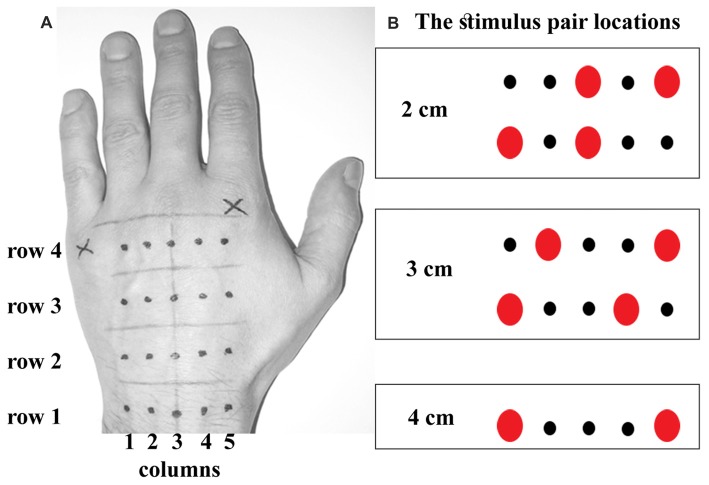
The stimulus locations. **(A)** shows the locations marked on the back of the participant’s hand. There were four rows in a grid, equally spaced apart. Each row contained five locations (dots) spaced 1 cm apart. **(B)** shows five possibilities for actual dual touch stimulation at each of four rows, rendering thus a total of *20 touch location pairs*. The dual touches were always delivered within a row, i.e., across the hand. Note a perfect symmetry in stimulation frequency between corresponding ulnar and radial sides on the hand. The 4 cm stimulus was doubled in number of trials to match the trial number of smaller distances.

The experimenter marked locations of tactile stimuli on the participant’s skin using light brown and black pens. The hand was occluded while the locations were drawn, and the participant could not see them until the experiment was complete. The first line was drawn across participant’s left wrist passing through a center of the ulnar styloid process. Another line running in parallel with the first line was drawn below a visible point of a separation between a pinkie and ring finger. Three intermediate lines were added at equal intervals, dividing the hand into four equally long subregions. The final line was drawn perpendicular to the center of a wrist-hand intersection. Four stimulus locations were marked along this line at the center of each subregion with a dark pencil. Two more locations spaced 1 cm apart were added to the right and left of each of these points at the longitudinal center of each subregion. This resulted in a grid consisting of four rows in proximo-distal orientation and five columns in ulnar-radial orientation (Figure 1A).

The experimenter then marked the apices of the knuckles of the index and little fingers with a hand clenched in a fist. Then, with the hand placed flat on a table, she recorded their distance as participant’s hand width, and photographed the hand. The distance between these knuckles was saved for a generic medium-sized male hand, the silhouette of which was later presented to participants to collect their responses in the tactile localization task. The size of a silhouette was automatically adjusted by a locked ratio in both dimensions until its width corresponded with a width of the participant’s hand.

A custom-written script was used to run the experiment (Cogent 2000 toolbox for Matlab 2015, Mathworks, Natick, MA, USA). At each trial, the experimenter would apply two simultaneous or sequential (1-s delay) touches across the hand at two of the marked locations using Von Frey filaments (60 g). Each touch lasted ~1 s. The stimulations were presented in two blocks per temporal delay manipulation counterbalanced across participants according to a Latin square. Each trial comprised of a stimulation pair 2 cm, 3 cm or 4 cm apart at one of the subregions along the hand. The 2 cm stimulations were applied with one touch at a central location and the other touch at one of the outer locations (Figure 1B). The 3 cm stimulations were at one of the outer locations and at the corresponding third point away across the hand. The 4 cm stimulations at the outer locations were doubled in number to compensate for a lack of alternative locations. All stimulations were randomized. There were 48 trials in each block. The half of sequential touches was presented in a reverse order of the locations. Thus, the blocks of sequential touches included randomized left-right and right-left stimulations.

To minimize experimenter error, the instructions were displayed on an image of a hand silhouette on the experimenter’s monitor. The background of a stimulated region was white against a gray background and the stimulation locations were red and larger compared to non-stimulated locations in black color. Any additional information including the order of sequential stimulations was presented in text above the image. After the stimuli were applied, the experimenter pressed the key on a keyboard for a hand silhouette and a mouse cursor to appear on the participant’s screen.

The silhouette image was a high contrast black and white picture rendering a white hand on a black background. The mouse cursor showed on participant’s screen as a thin cross at random positions on each trial. The participants indicated the location of each perceived touch by clicking the mouse cursor at the corresponding location on the silhouette hand. They were instructed to click twice at the same location when they perceived a single touch. The position of the mouse clicks was automatically recorded by the software. While their right hand moved in order to register the responses, participants were instructed not to move their left hand to minimize the use of proprioceptive information in the task. Participants were instructed to be precise in their responses and to avoid ballistic points. They were asked to report any accidental responses which were later removed from the analysis.

### Data Processing

The actual stimulus locations coded in *x* and *y* pixel coordinates on photographs of participants’ hands had to be aligned with the corresponding perceived locations marked on the size-matched hand silhouettes on the computer screen. Thus, the location of the knuckle of the little finger and that of the index finger on each photograph and hand silhouette pair were defined as points (0,0) and (1,0), respectively, with all other points assigned corresponding coordinates using Bookstein’s (1991) two-point registration method. Hand sizes for all participants were therefore normalized into a common body-scaled coordinate frame in which one Bookstein unit was defined as the distance between the knuckles of the index and little fingers. The coordinate axes were then aligned to correspond with the rows and columns of the stimulation grid.

Localization error was computed using the Bookstein *x* and *y* coordinates of the perceived and actual touches. A systematic localization bias (*constant* localization error) is a vector of a straight line between the actual and averaged perceived stimulus locations. The length of this vector represents the magnitude of the error in a certain direction while the angle relative to *x* axis represents the error direction. Here the analyses focused on the magnitude of constant error in two directions, one aligned with the hand’s proximo-distal axis and the other aligned with its ulnar-radial axis. These biases were determined from Bookstein coordinate values. Further, each set of localization attempts deviates from their average. The standard deviation computed across *x* and *y* coordinate values separately was used to quantify the *variable* error i.e., the precision of localization in ulnar-radial and proximo-distal orientation, respectively.

A critical distinction between the analyses of the single-touch localization error and the dual-touch localization error is that analyses concerning the latter must take into account that the dual touches are mislocalized *relative to each other*. To illustrate this, consider an example of two touches misplaced towards each other by 0.1 Bookstein units. Two observations can be made with the constant localization error quantified on the *x* axis aligned with the dual touch orientation. First, the sum of the localization error of 0.1 (radial) and −0.1 (ulnar) is zero, which as an average trend in the data would eliminate an overall translation of tactile perceptual space in ulnar or radial direction. Second, the relative mislocalization towards each other is given by a slope of a line fitted to these two values. A positive slope indicates that the touches were perceived as attracted while a negative slope indicates repulsion. Similarly, as an average trend in the data, the slope across ulnar-to-radial locations of the grid would indicate the scaling of the perceptual space given the symmetry in stimulation frequency across ulnar and radial locations of the grid (see Figure 1).

To reiterate, the tactile stimulus pairs were always applied within a row, i.e., *across* the hand. There were three different distances between dual touches which were applied in a manner aiming for a perfect symmetry in frequency of stimulations between the corresponding ulnar and radial sides of the grid. This was necessary in order to dissociate the factors of a distance manipulation and skin location which would have been impossible had we used the same two locations per distance. Clearly, the localization error could not be computed simply at each location of the grid which is customary in the single-touch analysis. Most locations of the grid would be used for different stimulus distances, and apart from 4 cm stimulus the same stimulus distance at any given proximo-distal region was a combination of different grid locations. Thus, the localization error was calculated for each touch of the 20 possible tactile stimulus pairs (five in each row; see Figure 1) in the first step, and then averaged depending on whether the analysis focused on stimulus distance or individual grid locations. The constant localization error and the variable localization error in two directions on the hand (proximo-distal and ulnar-radial) are presented in two separate parts of the “Results” section. Each section further breaks down to report the modulation of the localization error type by the stimulus distance and actual grid location. Their findings are complementary. For instance, the effects of the stimulus distances inevitably produce the findings which are not *location-specific*. They are thus complemented by the location-specific results found at individual grid locations which however do not inform about the *distance* separating the dual touches.

Finally, a false discovery rate Holm-Bonferonni procedure (HB-corr) was used to correct for increased type I error risk in multiple comparisons and the Greenhouse-Geisser correction (GG-corr) was applied when the variance sphericity was violated in repeated-measures ANOVA.

## Results

### Constant Localization Error

#### Constant Error at Individual Grid Locations

**Figure 2:**
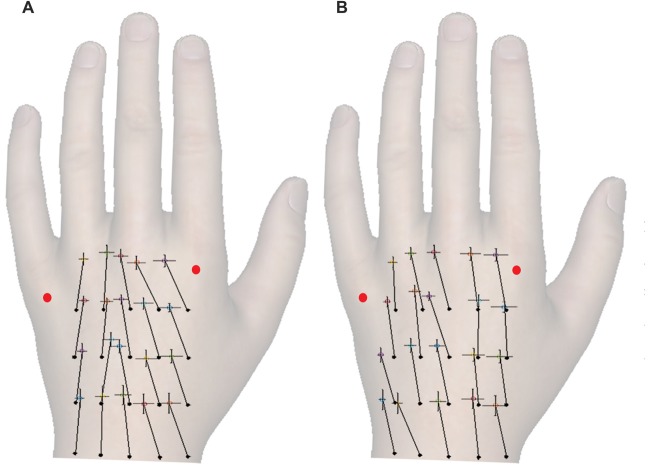
The constant localization error for different temporal separations of the touches. **(A,B)** show constant error for simultaneous and sequential touches, respectively. The full black circles mark the actual locations of tactile stimuli in a four-by-five grid, and the crosses show the corresponding perceived locations. The vector lengths and angles represent a magnitude and direction of constant localization error, respectively. Each cross additionally shows SEM of the mislocalization magnitude in ulnar-radial (0°) and proximo-distal (90°) orientation. The ulnar-radial axis is aligned to the grid and thus the wrist, not the knuckles. The red dots mark the knuckles. Although the responses were marked on a black and white hand silhouette, the hand image is used here for illustrative purposes.

Figures 2A,B shows the constant localization error for simultaneous and sequential touches. Relative to actual grid, the perceived locations were generally misplaced distally (M: 0.46, SD: 0.14), *t*_(24)_ = 16.97, *p* < 0.001, *d* = 3.39, which is consistent with the literature (Mancini et al., 2011; Margolis and Longo, 2015). However, contrary to the literature, the dual touches in this experiment were misplaced towards the ulnar edge of the hand (M: −0.04 Bookstein units, SD: 0.10), *t*_(24)_ = 2.42, *p* = 0.02, *d* = 0.48. A possible shift of the ulnar-radial center of the grid from under the middle finger towards the center given by a gap between the ring and middle fingers may be accountable for the overall ulnar shift.

**Figure 3:**
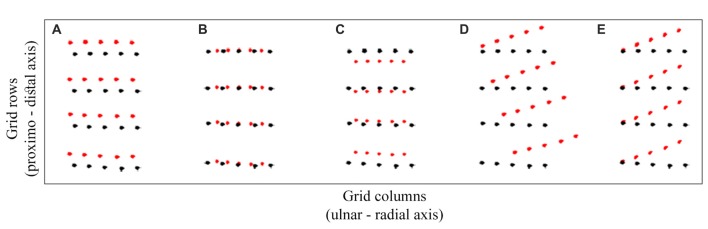
Transformation models. A few possibilities for a constant localization error patterns in the data are illustrated in **(A–E)**. The black circles represent the actual stimulus locations and the red circles represent the possible perceived locations. The displacement between the corresponding black and red circles is the constant localization error. The horizontal and vertical axes correspond with ulnar-radial and distal-proximal orientations on the hand. The panels show the **(A)** translation, **(B)** ulnar-radial compression, **(C)** overall compression, **(D)** orthogonal rotation and **(E)** non-orthogonal rotation, i.e., the skew of tactile perceptual space in relation to actual grid locations.

The dual stimuli were always applied within a row *across* the hand. To re-iterate, the same number of touches was applied to each corresponding ulnar and radial location (e.g., locations 1 and 5, and 2 and 4) irrespective of the stimulus distance. Moreover, to eliminate interference by the stimulus distance, the location possibilities for each distance were also balanced at the ulnar-radial grid center. This arrangement allowed us to assess, separately for the sequential and the simultaneous temporal presentation of the stimuli, any deviations in tactile perceptual space from the actual grid arrangement. Given that the judged and actual grid locations should in theory overlap, it is intuitive to think about the constant localization error in a context of translation, scaling, and rotation of perceptual space. Figure 3 shows a few simple illustrative examples of the constant localization error patterns indicative of the perceptual space *transformation*. Figure 3A shows the *translation* as an ulnar and distal shift of perceived (red) locations from their corresponding actual locations (black). As tactile pairs were always delivered across the hand, the funneling effect would show as a *compression* of perceptual space at the *x* axis, i.e., an increasing slope in ulnar bias across the ulnar-radial grid locations (Figure 3B). *Scaling* of both axes (Figure 3C) would show as a gradual increase in ulnar and distal error across grid locations away from a point of origin (e.g., gravitation towards the center of the grid). A *rotation* of perceptual space, e.g., by its alignment towards the line across knuckles, would also show as an interaction in constant localization error between grid rows and grid columns (Figures 3D,E).

To study the structure of perceptual space we conducted a repeated measures ANOVA. The constant localization error for two temporal separations (simultaneous and sequential touches) collapsed across different stimulus distances at each physical location of the grid was studied in two directions (ulnar-radial and proximo-distal), across grid rows (1–4; near wrist = 1, near knuckles = 4), and grid columns (1–5; most ulnar = 1, most radial = 5).

**Figure 4:**
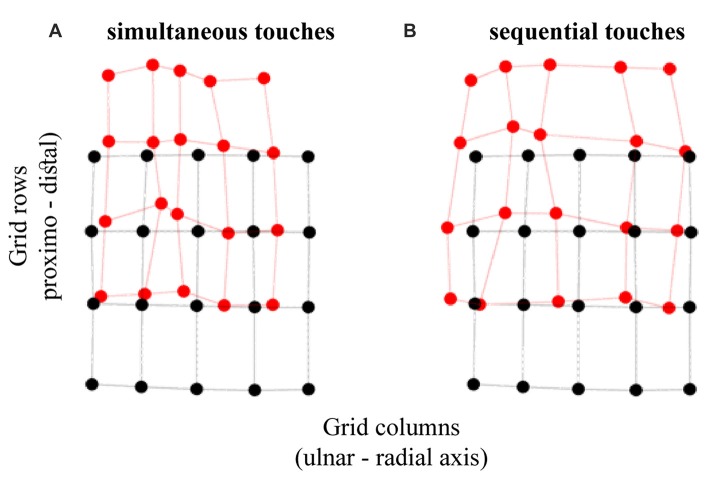
Tactile perceptual space in touch localization. The panels show the transformation of perceptual space relative to the configuration of actual grid for **(A)** simultaneous and **(B)** sequential dual touches. The black circles represent the actual stimulus locations and the red circles represent the average perceived locations. The displacement between the corresponding black and red circles is the constant localization bias. The horizontal and vertical axes correspond with ulnar-radial and distal-proximal orientations on the hand.

Unsurprisingly, the temporal separation of dual touches across the hand did not modulate, nor did it interact with other factors in modulating, the *distal* localization error (*p* > 0.40). The distal error did not change in magnitude across grid rows, *F*_(3,72)_ = 0.41, *p* = 0.67, $\eta_{\text{p}}^{2}$ = 0.02, or due to other factors interacting with this variable (*p* > 0.15). Without an interaction across grid rows and columns for both the distal and ulnar constant localization error we may rule out an overall scaling of the perceptual space and its rotation, e.g., towards the line across knuckles (Figures 3C–E). The distal mislocalization did differ across grid columns, *F*_(4,96)_ = 6.84, *p* < 0.001, $\eta_{\text{p}}^{2}$ = 0.22, by being larger for columns 2 and 3 (under the ring and middle finger; HB-corr *p* < 0.03). This is seen by a perceptual space warping at locations under the ring finger (Figure 4).

There were no differences in *ulnar* localization error across grid rows, *F*_(1.50,35.88)_ = 0.87, *p* = 0.40, $\eta_{\text{p}}^{2}$ = 0.04 (Greenhouse-Geisser corrected; GG-corr), but it increased with proximity to the thumb, *F*_(1.76,42.29)_ = 7.66, *p* = 0.002, $\eta_{\text{p}}^{2}$ = 0.24 (GG-corr). Intriguingly, there was an interaction between grid rows and columns, *F*_(6.12,146.86)_ = 3.05, *p* = 0.01, $\eta_{\text{p}}^{2}$ = 0.11 (GG-corr), which was not affected by temporal presentation of dual touches, *F*_(6.79,163.04)_ = 1.43, *p* = 0.20, $\eta_{\text{p}}^{2}$ = 0.06 (GG-corr). The interaction is driven by the distance between columns 2 and 3 being compressed more at central grid rows than near wrist and knuckles (*p* > 0.04). Given these localization bias patterns, we eliminated an overall scaling at both axes and the rotation of the perceptual space.

**Figure 5:**
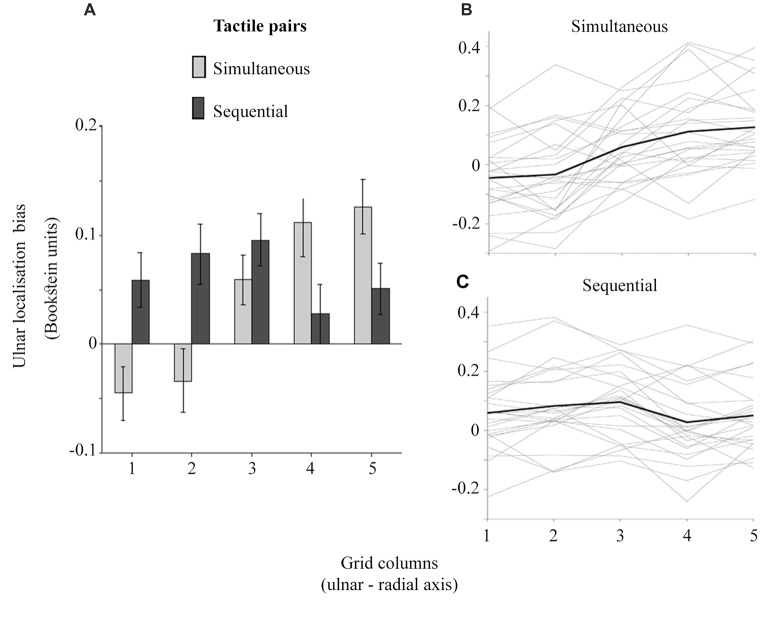
The ulnar localization error for simultaneous and sequential touches across grid columns. The larger grid column values are more proximal to the thumb. **(A)** shows the averaged localization error. The error bars are ± SEM. The inter-individual variability is plotted in **(B,C)**. The gray lines represent the slopes for each participant while the black line represents the averaged slope in each condition.

The ulnar localization error increasing with the proximity to thumb across grid columns attests to tactile perceptual space being compressed on the horizontal axis as would be expected with the funneling effect (Von Békésy, 1957; Chen et al., 2003). Figure 5 shows that the simultaneous touches, feeling closer to each other, produce larger opposite trends in ulnar bias compared to sequential touches. Accordingly, the ulnar bias was modulated by an interaction between temporal presentation of dual touches and their location across grid columns (Figure 5), *F*_(4,96)_ = 30.52, *p* < 0.001, $\eta_{\text{p}}^{2}$ = 0.56, but not grid rows, *F*_(1.98,47.63)_ = 1.51, *p* = 0.23, $\eta_{\text{p}}^{2}$ = 0.06 (GG-corr). A linear model fitted to the data across grid columns showed no slope in ulnar bias for the sequential stimuli (M: −0.01, SD: 0.03), *t*_(24)_ = 1.26, *p* = 0.22, *d*_z_ = 0.25, while the slope observed for the simultaneous touches (M: 0.05, SD: 0.04) was indeed increasing, corroborating thus the funneling effect in this condition, *t*_(24)_ = 5.94, *p* < 0.001, *d*_z_ = 1.19 (HB-corr). Compared to slope of sequential touches, the simultaneous stimuli were perceptually pulled towards each other, *t*_(24)_ = 8.13, *p* < 0.001, *d*_z_ = 1.63 (HB-corr).

Finally, one-sample *t*-tests showed that the ulnar bias is different from zero only at the columns 4 and 5 for the simultaneous touches, and 2 and 3 for the sequential touches (Holm-Bonferroni corrected *p* < 0.05). The larger ulnar bias at the radial locations for simultaneous touches is related to the perceptual space compression. Conversely, an expansion trend at the *x* axis is suggested by the *larger* ulnar bias at the ulnar side of the hand and a nearly veridical percept at radial locations for the sequential touches. The interpretation of these results becomes clearer when the *perceived distance*, i.e., the shortest line connecting any two localized touches is considered.

##### Perceived distance

The funneling effect is shown in the distance data, i.e., the straight line between each two perceived locations of a tactile stimulus pair. The distance is smaller for simultaneous touches compared to sequential touches, *F*_(1,29)_ = 68.30, *p* < 0.001, $\eta_{\text{p}}^{2}$ = 0.70. Relative to actual distance, there was an underestimation of perceived distance for touches presented simultaneously (M: −16.29%, SD: 31.88), *t*_(29)_ = 2.80, *p* = 0.01, *d*_z_ = 0.51, and an overestimation for the sequential touches (M: 28.77%, SD: 19.98), *t*_(29)_ = 7.89, *p* < 0.001, *d*_z_ = 1.44 (HB-corr). These results are complementary to an earlier reported compression, and a trend for expansion, respectively.

The strong overestimation for sequential touches is noteworthy as it occurred while there was no clear horizontal expansion of tactile perceptual space. This finding is clearer when interpreted with respect to the reported increase in *distal* mislocalization at grid columns 2 and 3, and the increase in *ulnar* mislocalization for sequential touches for the same grid columns. It seems that some responses may have been perceptually rotated relative to the actual orientation of the touches, which allowed to increase the distance separating them while being constrained by the actual hand size. Thus, while the straight-line distance between two touches was clearly increased, there was only a trend for the horizontal expansion of the perceptual space.

#### Constant Localization Error as a Function of Tactile Distance

Whereas the variations in constant localization error across individual grid locations enable the investigation of the tactile perceptual space, they disregard the effects of actual stimulus distances. Thus, the ulnar-radial and proximo-distal localization biases collapsed across different tactile locations for each stimulus distance were investigated in a repeated-measures ANOVA with the stimulus distance (2, 3 and 4 cm), temporal separation (simultaneous and sequential touches), and subregion along the hand (near wrist = 1, central = 2, 3, and near knuckles = 4). The latter represents the rows of the grid. All dual stimuli were applied in a single orientation *across* the hand at these subregions.

**Figure 6:**
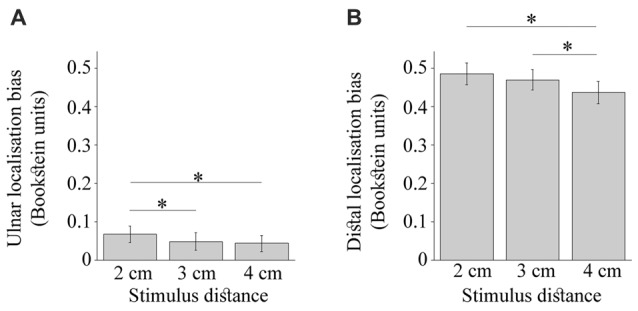
The constant localization bias modulated by the stimulus distance. **(A)** shows the *ulnar* bias to be largest for the 2 cm stimulus. **(B)** shows the *distal* bias to be smallest for 4 cm stimulus. The error bars are ± SEM. The Bookstein unit of 1 is the distance between the knuckles of the pinkie and the index finger normalizing thus across different hand sizes. The asterisks mark comparisons significant at *p* < 0.05.

As shown in Figure 6A, the bias towards the ulnar side of the hand was driven by larger displacement of 2 cm stimulus, *F*_(2,48)_ = 5.34, *p* = 0.01, $\eta_{\text{p}}^{2}$ = 0.18. The touches 2 cm apart were mislocalized more than those 3 cm apart, *t*_(24)_ = 2.70, *p* = 0.04, *d*_z_ = 0.54, and 4 cm apart, *t*_(24)_ = 2.66, *p* = 0.04, *d*_z_ = 0.53 (HB-corr). The distance separating dual touches also modulated the distal mislocalization (Figure 6B), *F*_(2,48)_ = 9.34, *p* < 0.001, $\eta_{\text{p}}^{2}$ = 0.28, which was reduced for 4 cm relative to 3 cm, *t*_(24)_ = 3.81, *p* = 0.003, *d*_z_ = 0.76, and 2 cm, *t*_(24)_ = 3.75, *p* = 0.003 *d*_z_ = 0.75 (HB-corr). Finally, the ulnar and distal localization bias were not different across grid rows or due to an interaction of this factor with other manipulated variables (*p* values > 0.10). Thus, the constant localization error in this study was not affected by the proximity of joint landmarks which is consistent with the literature (Mancini et al., 2011; Margolis and Longo, 2015).

No further modulation of the constant localization error was observed. The modulation of ulnar error (*p* = 0.88) and distal error (*p* = 0.53) by actual stimulus distance did not differ between simultaneous and sequential touches. There was a trend for the simultaneous touches to be displaced less towards the ulnar side of the hand compared to sequentially presented touches, *F*_(1,24)_ = 4.20, *p* = 0.05, $\eta_{\text{p}}^{2}$ = 0.15. As expected, the temporal separation did not modulate the distal error, *F*_(1,24)_ = 0.00011, *p* = 0.99, $\eta_{\text{p}}^{2}$ = 0.

### Variable Localization Error

#### Variable Error as a Function of Stimulus Distance

The ulnar-radial and proximo-distal *variable* localization error collapsed across different tactile locations for each stimulus distance were investigated in repeated-measures ANOVA with the stimulus distance (2, 3 and 4 cm), temporal separation (simultaneous and sequential touches), and subregion along the hand (near wrist = 1, central = 2, 3, and near knuckles = 4). The latter represent the rows of the grid. All dual stimuli were applied *across* the hand and within rows.

**Figure 7:**
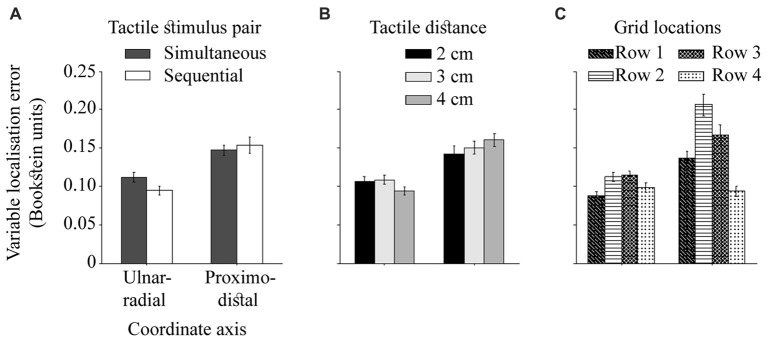
The ulnar-radial and proximo-distal variable localization error. The difference in variable error on two hand-centered coordinate axes as modulated by **(A)** temporal separation of dual touches, **(B)** spatial separation of dual touches, and **(C)** across grid rows (row 1 = near wrist). The precision is calculated for each axis as a standard deviation of individual localization attempts. The error bars are ± SEM.

The ulnar-radial variable error was reduced relative to proximo-distal variable error, *F*_(1,24)_ = 47.19, *p* < 0.001, $\eta_{\text{p}}^{2}$ = 0.66, as reported by Margolis and Longo (2015). This effect is presumably due to increased spatial precision of touch across the arm than along it (Boring, 1942; Schlereth et al., 2001; Cody et al., 2008), and/or additional clues of perceived tactile distance. The difference in variable error in the both directions was modulated by the temporal and spatial separation of dual touches (Figures 7A,B), *F*_(1,24)_ = 9.96, *p* < 0.001, $\eta_{\text{p}}^{2}$ = 0.29 and *F*_(2,48)_ = 4.24, *p* = 0.02, $\eta_{\text{p}}^{2}$ = 0.15, respectively. The ulnar-radial error was improved for touches presented with a delay compared to simultaneous touches, *t*_(24)_ = 2.50, *p* = 0.04, *d*_z_ = 0.50, whereas no difference due to temporal separation was observed for the proximo-distal error, *t*_(24)_ = 0.84, *p* = 0.41, *d*_z_ = 0.17 (HB-corr). Thus, the simultaneity of dual stimuli across the hand appears to make the localization task more difficult at the ulnar-radial axis alone. Similarly, the ulnar-radial localization of tactile pairs 4 cm apart is improved relative to those 3 cm apart, *t*_(24)_ = 2.73, *p* = 0.04, *d*_z_ = 0.55, and 2 cm apart, *t*_(24)_ = 2.40, *p* < 0.05, *d*_z_ = 0.48 (HB-corr). The actual locations of 4 cm in proximity of the hand edges may account for this improved localization.

Stimuli located near landmarks (wrist and knuckles) are localized better than stimuli in the middle of the hand (Figure 7C), *F*_(3,72)_ = 25.04, *p* < 0.001, $\eta_{\text{p}}^{2}$ = 0.51. The localization performance deteriorates at the row 2 relative to an adjacent wrist row 1, *t*_(24)_ = 4.80, *p* < 0.001, *d*_z_ = 0.96, and at row 3 relative to the adjacent knuckle row 4, *t*_(24)_ = 6.39, *p* < 0.001, *d*_z_ = 1.28 (HB-corr). The overall differences in performance between two central rows, *t*_(24)_ = 2.30, *p* = 0.03, *d*_z_ = 0.46 and the rows near wrist and knuckles, *t*_(24)_ = 3.33, *p* = 0.01, *d*_z_ = 0.67 (HB-corr) are driven by proximo-distal localization variability. While it is reduced near wrist compared to the knuckle area, *t*_(24)_ = 5.40, *p* < 0.001, *d*_z_ = 1.08, and at the row 2 compared to the row 3, *t*_(24)_ = 2.82, *p* = 0.03, *d*_z_ = 0.56, there is no difference in *ulnar-radial* variable error near wrist and knuckles, *t*_(24)_ = 1.78, *p* = 0.17, *d*_z_ = 0.36, and between the two central rows, *t*_(24)_ = 0.23, *p* = 0.82, *d*_z_ = 0.05 (HB-corr). The findings of an improved variable localization error near landmarks are consistent with the literature (Cholewiak and Collins, 2003).

#### Variable Error at Individual Grid Locations

The variable localization error for two temporal stimulus presentations (simultaneous and sequential touches), collapsed across different stimulus distances at each physical location of the grid, was investigated in two directions (ulnar-radial and proximo-distal coordinate axes), across grid rows (1–4; near wrist = 1, near knuckles = 4), and grid columns (1–5; most ulnar = 1, most radial = 5).

**Figure 8:**
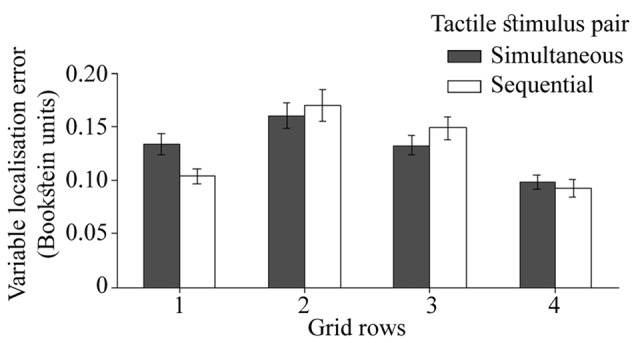
The localization precision (variable error) for two temporal separations of dual touches at each of the four rows on the hand. The ulnar-radial and proximo-distal precision is collapsed together. The error bars are SEM.

Although the interaction between the temporal separation of dual touches and proximo-distal stimulus locations did not reach significance in the analysis reported in previous section (*p* = 0.06), here the precision in localization of touches near the wrist (row 1) differed for simultaneous and sequential touches (Figure 8), *F*_(3,72)_ = 3.18, *p* = 0.03, $\eta_{\text{p}}^{2}$ = 0.12. Compared to sequentially presented touches, the localization attempts for simultaneous stimulations were more variable, *t*_(24)_ = 2.67, *p* = 0.01, *d*_z_ = 0.53. The 3-way interaction with the direction of variable localization error did not reach statistical significance, *F*_(3,72)_ = 2.02, *p* = 0.12, $\eta_{\text{p}}^{2}$ = 0.08 suggesting that the effect was not unique to one of the error directions.

## Discussion

The aim of this study was to complement and relate the two independent streams of literature, one investigating the systematic errors and precision in localization of single touches, and the other investigating tactile distance perception between dual touches. To this end, we studied constant and variable error in dual touch localization. Relative to their actual locations, the dual touches were displaced towards the fingers and the ulnar side of the hand. The ulnar mislocalization was modulated by temporal separation of tactile stimulus pairs. The touches presented simultaneously were mislocalized towards each other and as a result their distance was perceived to be smaller. A corresponding compression of tactile perceptual space in comparison with actual grid configuration is reported. The touches presented sequentially were mislocalized towards the ulnar side of the hand, with slightly increased bias and misjudgment of their orientation at more ulnar locations, which corresponded with overestimation of their distances. This resulted in a trend for expansion of tactile perceptual space. The variable localization error was reduced near joints, particularly near knuckles, while there were no near-joint differences in constant localization error. Further, it increased for the simultaneous touches and it was modulated by the stimulus distance.

### Dual Touch Localization and Tactile Perceptual Space

To guide the reader through the patterns of tactile mislocalization for a four-by-five stimulus grid on the hand dorsum, we constructed the perceptual map in a skin-centered spatial reference frame, using the constant localization error, i.e., the relative position of actual and perceived grid locations. We replicated the distal displacement of the perceptual map from the actual grid, which is associated with a supra-modal representation of body surface in single-touch localization literature (Mancini et al., 2011; Margolis and Longo, 2015). However, we did not replicate the radial localization bias (Mancini et al., 2011). It should be noted that the radial bias would be replicated with the ulnar-radial abscissa of the coordinate system across knuckles as reported by Mancini et al. (2011). However, rather than reflecting an overall radial shift of the perceived touches relative to their actual stimulus locations, the bias in our case would be a mathematical artifact of a misaligned coordinate frame relative to the stimulation grid. To illustrate this, consider an example in which each touch of a dual stimulus pair applied across hand is mislocalized distally, e.g., by 0.3 Bookstein units, while being localized accurately with relation to each other. The constant localization error computed within the rotated coordinate frame, e.g., anti-clockwise relative to stimulus orientation, would erroneously show for each touch a radial increase and distal decrease. Thus, the coordinate system should not be arbitrary in tactile localization studies and it should be aligned with the grid. The actual stimulus grid was aligned to knuckles in the study by Mancini et al. (2011), while it is aligned with the wrist in this study. With our grid and coordinate frame aligned, the ulnar bias represents a genuine displacement of the perceived touches towards the ulnar hand side.

A separate issue is a rotation of the perceptual map relative to the orientation of the actual stimulus grid. Such misalignment would for instance occur if participants were to mark their responses as tilted with reference to knuckle landmarks in our experiment. However, we found no evidence for the rotation of tactile perceptual space relative to the orientation of actual stimulus grid. This finding is important in two respects. First, we may conclude an overall accuracy in perceived orientation of the dual touches on the hand dorsum which suggests the skin-centered tactile judgments. Second, the knuckle landmarks, playing a significant role in the distal displacement of touches but not explaining it fully (Margolis and Longo, 2015), do not contribute to tactile mislocalization by creating a misalignment angle for the perceptual map relative to actual grid. This includes the non-orthogonal rotation which would not preclude the accurate stimulus orientation. Nevertheless, we recommend that the future single-touch localization studies further investigate the perceptual map rotation without the clues given by an orientation of dual stimuli in this study.

The tactile stimuli were presented across the hand with the size-matched hand silhouette presented visually on a computer screen each time the response was given. However, the distances separating the sequentially presented touches were overestimated regardless of the veridical information about the hand size. This finding is accompanied by a larger ulnar bias in tactile localization at the ulnar side of the hand compared to locations near thumb, while the distal bias doesn’t vary across grid rows. Together these results would suggest an expansion of the perceptual space consistent with the squatter and shorter perceptual maps of the hand dorsum (Longo and Haggard, 2010; Longo and Golubova, 2017). However, the present data cannot rule out a possible scaling at the *y* axis with an inclusion of proximo-distally oriented dual touches, be it a compression and thus the shorter and squatter perceptual map, or an expansion matching or exceeding that in the ulnar-radial direction. Thus, only the *relative* somatotopic activation for dual touches resulted in a transformation of tactile perceptual space. No such influence is found when the relative position is not computed in the proximo-distal orientation in this experiment.

The availability of information about veridical hand size, provided prior each response by a size-matched hand silhouette on a computer screen, did not prevent the compression of tactile perceptual space at the ulnar-radial axis, and the perceived compression of stimulus distances. Von Békésy (1975) discussed the summation and inhibition of tactile signals when he observed the perceived distance monotonously increasing from zero (tactile spatial acuity threshold for given skin region) with the increasing actual distance. This was corroborated in Weber’s illusion studies where the same distance feels smaller on less sensitive skin regions with higher acuity thresholds (Taylor-Clarke et al., 2004; Anema et al., 2008; Miller et al., 2016). Accordingly, we report a compressed map for simultaneous stimulations on the hand dorsum, given that the threshold for the dorsum is ~1.6 to 2 cm (Weinstein, 1968; Mancini et al., 2014), and we used 2 cm, 3 cm and 4 cm stimuli. However, the influence of these low-level factors is reduced with increased temporal separation between the touches. In the Tau effect (Helson, 1930; Jones and Huang, 1982; Goldreich, 2007), a person given two successive spatial intervals, automatically equalizes the ratios of their temporal presentation with the ratios of their perceived distance. Thus, the perceived spatial separation may increase due to increased temporal separation. In our study, however, the simultaneous and sequential stimulations were presented in separate blocks of 48 trials each, which rules out their relative comparison characteristic for Tau effect experiments. A compression on the arm in dual-touch localization was also reported by Green (1982) while the single touches were mislocalized towards the joints. It was posited that the single touches were localized with respect to body as reference frame whereas the dual touches were mislocalized with respect to each other (Boring, 1942; Green, 1982). Our results are consistent with this interpretation. The simultaneous dual touches localized with reference to each other were subject to influence of low-level somatosensory factors. Conversely, the localization of sequential touches may have become more like that of single touches using the hand as reference frame (Green, 1982).

Although we acknowledge the role of peripheral factors in our interpretation, we do not posit that the observed perceptual compression may be explained purely by the summation and inhibition of tactile inputs in the periphery and S1. In the related tactile distance perception literature, distortions of perceived distance between two touches are substantially diminished relative to what would be expected based on magnification factors, innervation density and RF properties (Taylor-Clarke et al., 2004; Longo, 2017). The “top-down” influences and a secondary body representation which reduce these early distortions are widely discussed in the literature (Taylor-Clarke et al., 2004; Linkenauger et al., 2015; Longo, 2017). Indeed, the inhibitory interneuron connections at different levels of somatosensory processing may be both feedforward and feedback, which allows for top-down modulation e.g., by attention (Gardner and Johnson, 2000). Given that we do not think it is possible to disentangle the peripheral and central factors in a context of this study, we refrain from a firm attribution of the observed effects to either one of them alone.

Finally, the overall translation in the ulnar direction was larger for the sequential touches. To reiterate, we used a balanced design in which the randomized trials were presented, half in the right-left and the other half in the left-right direction. While the order of stimulations was not an independent variable in this study, future research should address the effects of sequential stimulus direction in the translation of perceptual space. The ulnar shift of perceptual space, however, is likely to be a result of shifted perceived central location of the column 3 from under the middle finger towards the gap between the middle and ring fingers. Consistent with our interpretation concerning the increased use of hand reference frame in this condition, this re-centering of the column 3 would have had greater impact in localization of sequential touches.

We additionally reported a reduced ulnar-radial *variable localization error* compared to that on the proximo-distal axis. It may be due to spatial sensitivity differences attributable to oval-shaped neuronal RFs on the hand dorsum (Longo and Haggard, 2011; Longo and Golubova, 2017), or simply due to additional clues of more than one touch in this direction. Unsurprisingly, the ulnar-radial precision deteriorates for simultaneous touches which make the task more difficult while the proximo-distal precision remains unchanged for them. Further, the overall variable error at both axes for the funneled stimuli is larger near wrist relative to knuckles while being smaller from that at the center of the hand. The variable error for sequential touches shows a similar improvement near both joint landmarks. Taken together, these findings show a clear dissociation between the constant and variable localization errors, and they highlight the role of skeletal landmarks in tactile localization.

### The Role of Joints/Skeletal Landmarks in Tactile Localization

We replicated the constant localization error in a distal direction reported in single-touch studies (Mancini et al., 2011; Margolis and Longo, 2015). The literature highlights a role of perceived *knuckle* locations given that the bias is partly reduced with their view (Margolis and Longo, 2015). The evidence from this study shows that the distal bias is also reduced at all regions along the hand for the largest 4 cm distance of dual touches. This reduction cannot be attributed to the effects of lateral inhibition given its similarity across simultaneously and sequentially presented touches. Whereas the proximo-distal precision did not differ across stimulus distances, the ulnar-radial precision was improved for 4 cm stimuli at each grid row possibly due to the fact that on 4 cm trials, both touches always occur contiguous to the hand edges. This interpretation is compatible with the greater ulnar-radial precision observed at all outer ulnar-radial locations of the grid irrespective of applied stimulus distances. Moreover, the 4 cm stimulus was applied at the same ulnar-radial locations unlike the smaller stimuli which could be shifted one or two locations to the side. Thus, at least for the hands, a displacement of the perceived touch further distally is associated with the less distinct touches which are also localized with a poorer precision.

The reduced distal constant bias for touches which are localized with better precision, i.e., reduced variable error, is consistent with a smaller distal bias for a more precisely localized touch on more sensitive palm of the hand (Mancini et al., 2011), and with both the constant and variable bias being smaller in ulnar-radial orientation on the hand dorsum (Mancini et al., 2011; Margolis and Longo, 2015). However, improved localization precision does not automatically imply a reduction in the constant bias. Whereas there was a superior localization precision near knuckles (row 4) relative to all other rows, the evidence does not show for the knuckle landmarks to play a special role in modulating the distal localization bias at this region more than elsewhere on the hand. This finding is consistent with the evidence from single-touch localization studies, where it is interpreted with respect to constant localization bias being associated more closely with the secondary body representation while the variable error is thought to reflect more the somatotopic factors (Mancini et al., 2011; Margolis and Longo, 2015).

Our data suggests that the enhanced precision near knuckles is due to proximo-distal precision being greater than that observed at other regions. Precision in the proximo-distal axis is also improved near the wrist compared to central regions of the dorsal hand, and the radial-ulnar precision for the joint boundary regions is similar and greater compared to that at the central regions. Thus, although the near-joint proprioceptive information (Green, 1982; Cholewiak and Collins, 2003) and perhaps greater sensitivity to skin stretch (Edin and Abbs, 1991; Edin, 1992, 2004; Edin and Johansson, 1995) may have improved the localization precision near both joint boundaries, the precision in proximo-distal direction was enhanced less near the wrist than near the knuckles, presumably due to the overall constant localization error in distal direction away from the wrist. The distal shift near the wrist is surprising given that the shape of the hand with its characteristic wrist narrowing and the location of the thumb is clear on a silhouette of the hand. One possibility that should be addressed in future localization studies is the weighting of the wrist landmark relative to a (possibly misperceived) knuckle location of the thumb.

Final consideration should be given to transfer between visual and tactile space which is a concern in most tactile localization tasks reported in the literature. Indeed, the participants viewed the hand silhouette to give the responses which may have affected their judgments in this study. However, even where there is not a visual-tactile transfer, some transfer between frames of reference is needed to obtain the responses at all. The study of Mancini et al. (2011), which introduced the paradigm we use here, attempted to deal with this concern in a “haptic variant” of the task, in which participants indicated perceived locations of touches on a rubber hand without the vision. However, even that haptic task isn’t a pure measure of tactile localization since it involves processes related to haptic exploration. The correspondence between the silhouette task and the haptic task, however, does suggest that the basic pattern of biases is not due to idiosyncratic aspects of either methods.

In summary, the distal bias reported in the literature is consistent for locations marked on a hand silhouette and those pointed at in corresponding locations of a prosthetic rubber hand (Mancini et al., 2011) indicating that the bias is not specific to the visual response modality. Further, the distal bias was reduced but not eliminated with the responses marked on hand photographs which ruled out the knuckle location misperception as a sole causal factor (Margolis and Longo, 2015). We reported an additional factor of a distance between dual touches to modulate the distal bias on the dorsal hand. We additionally reported a reduced variable localization error for dual touches near joints of the dorsal hand, particularly near the knuckle landmarks which are likely to be perceptually filled in on a hand silhouette image. This evidence is consistent with previous literature suggesting the enhanced near-joint tactile localization due to additional proprioceptive signals (Green, 1982; Cholewiak and Collins, 2003). However, in our design, this evidence also underlines the combined influence of somatosensation and mental imagery concerning the perceived landmark locations.

## Conclusion

Taken together, the tactile localization literature suggests that the simultaneous dual touches too close in space cannot be discriminated as separate events but their detection is improved due to signal summation and inhibition (Von Békésy, 1975; Chen et al., 2003). The evidence shows that when they are separated sufficiently, they still cannot be accurately localized (Green, 1982) due to low-level factors including the innervation density and RF properties. By increasing their temporal separation, however, we found that these distorting low-level influences were alleviated. We interpreted our findings as a tendency to localize simultaneous dual touches with reference to each other being thus subject to influence of low-level somatosensory factors. Conversely, the localization of sequential touches in our experiment may have become qualitatively different, i.e., more like that of single touches localized with respect to body as a reference frame (Boring, 1942; Green, 1982).

Our findings are relevant to a current model for somatoperception being mediated by secondary body representation which alleviates the influence of the distorted somatotopic maps in SI (Longo et al., 2010; Medina and Coslett, 2010; Longo, 2015). While the distance perception literature suggests a top-down influences of body representation, which improve the somatoperception by alleviating the influence of distorted somatotopic maps, we report the effects of low-level factors concerning the organization of somatosensory system. Critically, the perceptual errors were not eliminated in a design where participants saw the hand silhouette matched in size to their hand each time they gave their response, suggesting a persistent bottom-up influence.

The aim of this study was to relate the findings from two predominantly separate streams of literature, one reporting on the localization of single touches, and the other on the distance perception of dual touches. The evidence showed that the dual touches, akin to single touch stimulations, were mislocalized distally. This effect was previously associated with a supra-modal representation of body surface (Mancini et al., 2011). Also consistent with the single-touch localization literature, the variable localization error is reduced near joints, particularly near knuckles. Finally, contrary to single-touch literature, we observed for the dual touches to be mislocalized towards the ulnar side of the hand. This displacement was larger for touches presented in sequential order than the simultaneous touches.

## Author Contributions

RS, LT, EW and MRL co-designed the experiment, and made contributions through the process of data collection to the manuscript finalization. RS was in lead of data analysis, writing and revising of the manuscript.

## Conflict of Interest Statement

The authors declare that the research was conducted in the absence of any commercial or financial relationships that could be construed as a potential conflict of interest. The reviewer LC and handling Editor declared their shared affiliation, and the handling Editor states that the process nevertheless met the standards of a fair and objective review.

## References

[B1] Anema H. A. van Zandvoort M. J. E. de Haan E. H. F. Kappelle L. J. de Kort P. L. M. Jansen B. P. W.. (2009). A double dissociation between somatosensory processing for perception and action. Neuropsychologia 47, 1615–1620. 10.1016/j.neuropsychologia.2008.11.001 19038277

[B2] Anema H. A. Wolswijk V. W. Ruis C. Dijkerman H. C. (2008). Grasping Weber’s illusion: the effect of receptor density differences on grasping and matching. Cogn. Neuropsychol. 25, 951–967. 10.1080/02643290802041323 18608322

[B3] Angel A. (1969). The central control of sensory transmission and its possible relation to reaction time. Acta Psychol. 30, 339–357. 10.1016/0001-6918(69)90058-4 5811526

[B4] Barghout A. Cha J. Saddik A. E. Kammerl J. Steinbach E. (2009). “Spatial resolution of vibrotactile perception on the human forearm when exploiting funneling illusion,” in Proceedings of IEEE International Workshop on Haptic Audio Visual Environments and Games, HAVE 2009, (Lecco, Italy), 19–23. Available online at: http://ieeexplore.ieee.org/xpl/mostRecentIssue.jsp?punumber=5351157

[B55] Bookstein F. L. (1991). Morphometric Tools for Landmark Data: Geometry and Biology. Cambridge, UK: Cambridge University Press.

[B5] Boring E. G. (1942). Sensation and Perception in the History of Experimental Psychology. New York, NY: Irvington Publishers.

[B6] Brown P. B. Koerber H. R. Millecchia R. (2004). From innervation density to tactile acuity: 1. Spatial representation. Brain Res. 1011, 14–32. 10.1016/j.brainres.2004.03.009 15140641

[B7] Chen L. M. Friedman R. M. Roe A. W. (2003). Optical imaging of a tactile illusion in area 3b of the primary somatosensory cortex. Science 302, 881–885. 10.1126/science.1087846 14500850

[B8] Cholewiak R. W. Collins A. A. (2003). Vibrotactile localization on the arm: effects of place, space, and age. Percept. Psychophys. 65, 1058–1077. 10.3758/bf03194834 14674633

[B9] Cody F. W. J. Garside R. A. D. Lloyd D. Poliakoff E. (2008). Tactile spatial acuity varies with site and axis in the human upper limb. Neurosci. Lett. 433, 103–108. 10.1016/j.neulet.2007.12.054 18255231

[B10] Edin B. B. (1992). Quantitative analysis of static strain sensitivity in human mechanoreceptors from hairy skin. J. Neurophysiol. 67, 1105–1113. 10.1152/jn.1992.67.5.1105 1597700

[B11] Edin B. B. (2004). Quantitative analyses of dynamic strain sensitivity in human skin mechanoreceptors. J. Physiol. 531, 3233–3243. 10.1152/jn.00628.2004 15548636

[B12] Edin B. B. Abbs J. H. (1991). Finger movement responses of cutaneous mechanoreceptors in the dorsal skin of the human hand. J. Neurophysiol. 65, 657–670. 10.1152/jn.1991.65.3.657 2051199

[B13] Edin B. B. Johansson N. (1995). Skin strain patterns provide kinaesthetic information to the human central nervous system. J. Physiol. 487, 243–251. 10.1113/jphysiol.1995.sp020875 7473253 PMC1156613

[B14] Förderreuther S. Sailer U. Straube A. (2004). Impaired self-perception of the hand in complex regional pain syndrome (CRPS). Pain 110, 756–761. 10.1016/j.pain.2004.05.019 15288417

[B15] Gardner E. P. Costanzo R. M. (1980). Temporal integration of multiple-point stimuli in primary somatosensory cortical receptive fields of alert monkeys. J. Neurophysiol. 43, 444–468. 10.1152/jn.1980.43.2.444 6770054

[B16] Gardner E. P. Johnson K. O. (2000). “Touch,” in Principles of Neural Science, 5th Edn. eds Kandell E. R. Shwartz J. H. Jessell T. M. (New York, NY: McGraw-Hill, Health Professions Division), 498–527.

[B56] Gardner E. P. Spencer W. A. (1972). Sensory funneling. I. Psychophysical observations of human subjects and responses of cutaneous mechanoreceptive afferents in the cat to patterned skin stimuli. J. Neurophysiol. 35, 925–953. 10.1152/jn.1972.35.6.925 4654255

[B17] Geldard F. A. Sherrick C. E. (1972). The cutaneous “rabbit”: a perceptual illusion. Science 178, 178–179. 10.1126/science.178.4057.178 5076909

[B18] Goldreich D. (2007). A Bayesian perceptual model replicates the cutaneous rabbit and other tactile spatiotemporal illusions. PLoS One 2:e333. 10.1371/journal.pone.0000333 17389923 PMC1828626

[B19] Green B. G. (1982). The perception of distance and location for dual tactile pressures. Percept. Psychophys. 31, 315–323. 10.3758/bf03202654 7110884

[B20] Harris J. A. Karlov L. Clifford C. W. G. (2006). Localization of tactile stimuli depends on conscious detection. J. Neurosci. 26, 948–952. 10.1523/JNEUROSCI.4318-05.2006 16421314 PMC6675354

[B21] Harris J. A. Thein T. Clifford C. W. G. (2004). Dissociating detection from localization of tactile stimuli. J. Neurosci. 24, 3683–3693. 10.1523/JNEUROSCI.0134-04.2004 15071117 PMC6729752

[B22] Head H. Holmes G. (1911). Sensory disturbances from cerebral lesions. Brain 34, 102–254. 10.1093/brain/34.2-3.102

[B23] Helson H. (1930). The Tau effect—an example of psychological relativity. Science 71, 536–537. 10.1126/science.71.1847.536 17799065

[B24] Johansson R. S. Vallbo A. B. (1979). Tactile sensibility in the human hand: relative and absolute densities of four types of mechanoreceptive units in glabrous skin. J. Physiol. 286, 283–300. 10.1113/jphysiol.1979.sp012619 439026 PMC1281571

[B25] Johansson R. S. Vallbo A. B. Westling G. (1980). Thresholds of mechanosensitive afferents in the human hand as measured with von Frey hairs. Brain Res. 184, 343–351. 10.1016/0006-8993(80)90803-3 7353160

[B26] Johnson K. O. (2001). The roles and functions of cutaneous mechanoreceptors. Curr. Opin. Neurobiol. 11, 455–461. 10.1016/s0959-4388(00)00234-8 11502392

[B27] Jones B. Huang Y. L. (1982). Space-time dependencies in psychophysical judgment of extent and duration: algebraic models of the tau and kappa effects. Psychol. Bull. 91, 128–142. 10.1037/0033-2909.91.1.128

[B28] Kaas J. H. Nelson R. J. Sur M. Lin C. S. Merzenich M. M. (1979). Multiple representations of the body within the primary somatosensory cortex of primates. Science 204, 521–523. 10.1126/science.107591 107591

[B29] Linkenauger S. A. Wong H. Y. Geuss M. Stefanucci J. K. McCulloch K. C. Bülthoff H. H.. (2015). The perceptual homunculus: the perception of the relative proportions of the human body. J. Exp. Psychol. Gen. 144, 103–113. 10.1037/xge0000028 25494548

[B30] Longo M. R. (2015). Implicit and explicit body representations. Eur. Psychol. 20, 6–15. 10.1027/1016-9040/a000198

[B31] Longo M. R. (2017). Distorted body representations in healthy cognition. Q. J. Exp. Psychol. 70, 378–388. 10.1080/17470218.2016.1143956 26823038

[B35] Longo M. R. Azañón E. Haggard P. (2010). More than skin deep: body representation beyond primary somatosensory cortex. Neuropsychologia 48, 655–668. 10.1016/j.neuropsychologia.2009.08.022 19720070

[B32] Longo M. R. Golubova O. (2017). Mapping the internal geometry of tactile space. J. Exp. Psychol. Hum. Percept. Perform. 43, 1815–1827. 10.1037/xhp0000434 28967785

[B33] Longo M. R. Haggard P. (2010). An implicit body representation underlying human position sense. Proc. Natl. Acad. Sci. U S A 107, 11727–11732. 10.1073/pnas.1003483107 20547858 PMC2900654

[B34] Longo M. R. Haggard P. (2011). Weber’s illusion and body shape: anisotropy of tactile size perception on the hand. J. Exp. Psychol. Hum. Percept. Perform. 37, 720–726. 10.1037/a0021921 21480744

[B57] Longo M. R. Sadibolova R. (2013). Seeing the body distorts tactile size perception. Cognition 126, 475–481. 10.1016/j.cognition.2012.11.013 23313871

[B36] Mancini F. Bauleo A. Cole J. Lui F. Porro C. A. Haggard P.. (2014). Whole-body mapping of spatial acuity for pain and touch. Ann. Neurol. 75, 917–924. 10.1002/ana.24179 24816757 PMC4143958

[B37] Mancini F. Longo M. R. Iannetti G. D. Haggard P. (2011). A supramodal representation of the body surface. Neuropsychologia 49, 1194–1201. 10.1016/j.neuropsychologia.2010.12.040 21199662

[B38] Margolis A. N. Longo M. R. (2015). Visual detail about the body modulates tactile localisation biases. Exp. Brain Res. 233, 351–358. 10.1007/s00221-014-4118-3 25300963

[B39] Medina J. Coslett H. B. (2010). From maps to form to space: touch and the body schema. Neuropsychologia 48, 645–654. 10.1016/j.neuropsychologia.2009.08.017 19699214 PMC2813960

[B40] Medina J. Coslett H. B. (2016). What can errors tell us about body representations? Cogn. Neuropsychol. 33, 5–25. 10.1080/02643294.2016.1188065 27386744 PMC5398312

[B41] Miller L. E. Longo M. R. Saygin A. P. (2016). Mental body representations retain homuncular shape distortions: evidence from Weber’s illusion. Conscious. Cogn. 40, 17–25. 10.1016/j.concog.2015.12.008 26741857

[B42] Oldfield R. C. (1971). The assessment and analysis of handedness: the Edinburgh inventory. Neuropsychologia 9, 97–113. 10.1016/0028-3932(71)90067-4 5146491

[B43] Penfield W. Boldrey E. (1937). Somatic motor and sensory representation in the cerebral cortex of man as studied by electrical stimulation. Brain 60, 389–443. 10.1093/brain/60.4.389

[B44] Rapp B. Hendel S. K. Medina J. (2002). Remodeling of somotasensory hand representations following cerebral lesions in humans. Neuroreport 13, 207–211. 10.1097/00001756-200202110-00007 11893911

[B45] Schlereth T. Magerl W. Treede R. D. (2001). Spatial discrimination thresholds for pain and touch in human hairy skin. Pain 92, 187–194. 10.1016/s0304-3959(00)00484-x 11323139

[B46] Sereno M. I. Huang R.-S. (2006). A human parietal face area contains aligned head-centered visual and tactile maps. Nat. Neurosci. 9, 1337–1343. 10.1038/nn1777 16998482

[B47] Sur M. Merzenich M. M. Kaas J. H. (1980). Magnification, receptive-field area and “hypercolumn” size in areas 3b and 1 of somatosensory cortex in owl monkeys. J. Neurophysiol. 44, 295–311. 10.1152/jn.1980.44.2.295 7411189

[B48] Tamè L. Pavani F. Papadelis C. Farnè A. Braun C. (2015). Early integration of bilateral touch in the primary somatosensory cortex. Hum. Brain Mapp. 36, 1506–1523. 10.1002/hbm.22719 25514844 PMC6869154

[B49] Taylor-Clarke M. Jacobsen P. Haggard P. (2004). Keeping the world a constant size: object constancy in human touch. Nat. Neurosci. 7, 219–220. 10.1038/nn1199 14966526

[B50] Von Békésy G. (1957). Neural volleys and the similarity between some sensations produced by tones and by skin vibrations. J. Acoust. Soc. Am. 29, 1059–1069. 10.1121/1.1908698

[B51] Von Békésy G. (1958). Funneling in the nervous system and its role in loudness and sensation intensity on the skin. J. Acoust. Soc. Am. 30, 399–412. 10.1121/1.1909626

[B52] Von Békésy G. (1975). Sensory Inhibition. Princeton, NJ: Princeton University Press.

[B53] Weber E. H. (1996). “De subtilitate tactus,” in The Sense of Touch, 2nd Edn, eds Ross H. E. Murray D. J. (East Sussex, UK: Erlbaum Taylor and Francis), (Original work published 1834) 21-128.

[B54] Weinstein S. (1968). “Intensive and extensive aspects of tactile sensitivity as a function of body part, sex and laterality,” in The Skin Senses, ed. Kenshalo R. S. (Springfield, IL: Thomas), 195–222.

